# The clinical features and outcomes of aggressive large B cell lymphoma with concomitant hemophagocytic lymphohistiocytosis at diagnosis

**DOI:** 10.1007/s00277-025-06574-w

**Published:** 2025-08-30

**Authors:** Lixia Zhu, Mengqi Xiong, Zixi Wang, Li Li, Jingsong He, Lijun Wang, He Huang, Xiujin Ye

**Affiliations:** 1 Bone Marrow Transplantation Center, The First Affiliated Hospital, Zhejiang University School of Medicine, 79# Qingchun Road, Hangzhou, 310003 China; 2 Liangzhu Laboratory, Zhejiang University Medical Center, Hangzhou, China; 3 Institute of Hematology, Zhejiang University, Hangzhou, China; 4 Zhejiang Province Engineering Laboratory for Stem Cell and Immunity Therapy, Hangzhou, China; 5 Department of Pathology, The First Affiliated Hospital, Zhejiang University School of Medicine, Hangzhou, China

**Keywords:** Hemophagocytic lymphohistiocytosis, Epstein–Barr virus, B-cell lymphoma, Ruxolitinib, Etoposide

## Abstract

Hemophagocytic lymphohistiocytosis (HLH), as a life-threatening hyperinflammatory syndrome, rarely presents as a harbinger of aggressive large B cell lymphoma (LBCL), with a rapidly progressive clinical course and poor prognosis. A total of 30 patients diagnosed with aggressive LBCL concurrent with HLH were retrospectively reviewed in this study. Median age was 60 years (range, 24 to 85 years). Thirteen (43.3%) patients treated with ruxolitinib combined with corticosteroid (Ru-D) regimen achieved the highest overall response rate (ORR) of 84.6%, which was significantly higher than that of 40.0% in the etoposide and corticosteroid group and 33.3% in the corticosteroid group (*P* = 0.019). The median overall survival (OS) was 16.2 months, with corresponding 1-year and 2-year OS rates of 63.3% and 38.4%, respectively. The 8-week mortality rate was 26.7%. Patients responded to anti-HLH therapy within 2 weeks had significantly better OS than non-responsive group (*P* = 0.009). Low-intensity chemotherapy without anthracycline as the first-line of anti-lymphoma therapy followed by RCHOP did not compromise survival, and the median OS was 13 months and 19.1 months, respectively (*P* = 0.457). Ferritin levels ≥ 3606 ng/mL and uncontrolled HLH within 2 weeks were the independent risk factors associated with inferior OS. Our findings highlight the high early mortality and short survival of these patients and underscore the urgent need for developing more effective treatment strategies to improve prognosis.

## Introduction

Hemophagocytic lymphohistiocytosis (HLH) is a life-threatening hyperinflammatory syndrome characterized by uncontrolled activation and proliferation of T-cells and macrophages. HLH can be either caused by genetic mutations, or secondary to various triggers such as malignancies, autoimmune disorders, or infections. Malignancy, especially lymphoma, is the most common etiology of HLH among adults [1, 2]. Lymphoma-associated HLH (LAHS) has a poor prognosis, with a median overall survival (OS) of less than 2 months in previous retrospective studies [3, 4]. HLH is commonly associated with T-cell and NK-cell lymphoma [5]. HLH also presents as a precursor to aggressive large B cell lymphoma (LBCL) in certain rare conditions [6–10].

In the era of rituximab, the treatment of diffuse large B-cell lymphoma has made a milestone leap, with approximately 50%−60% of patients achieving complete cure [11]. However, based on limited literature and clinical experience, LBCL concurrent with HLH at initial diagnosis often exhibits aggressive behavior, rapid progression, and poor prognosis. LBCL concurrent with HLH is often observed in patients who are EBV-DNA positive or have primary lymphoma involvement in bone marrow [6, 10]. Patients with HLH as the initial manifestation often exhibit concomitant cytopenia and hypofibrinogenemia, which increases the risk of invasive procedures and potentially delay the diagnosis of lymphoma. Therefore, early control of hemophagocytosis may be beneficial for timely diagnosis and prompt treatment of lymphoma. In addition, the timing and regimen of anti-HLH and anti-lymphoma therapy remain uncertain, and no recommended treatment strategies has been established.

Due to the rarity of this disorder, there are limited reported cases available, thereby underscoring the necessity of large-scale series studies. In the present study, we retrospectively investigated 30 patients diagnosed with aggressive LBCL presenting with HLH as the initial clinical manifestation, aiming to profile clinical characteristics, outcomes, and prognosis factors of this cohort.

## Methods

### Patients cohort

**Fig. 1:**
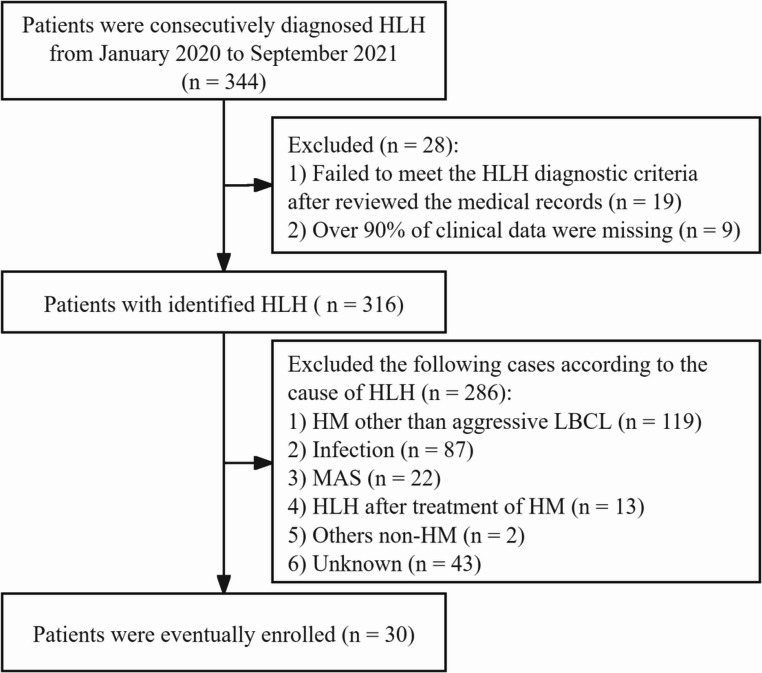
The flowchart for the screening of enrolled patients. HLH hemophagocytic lymphohistiocytosis, HM hematological malignancy, LBCL large B cell lymphoma, MAS macrophage activation syndrome

The present investigation was a single-center, retrospective study. Data from 30 patients newly diagnosed with aggressive LBCL accompanied by HLH in the First Affiliated Hospital of Zhejiang University between January 2020 and September 2022 were included in this study. Inclusion criteria were as follows: fulfillment of the HLH-2004 diagnostic criteria [12]; diagnosed with LBCL according to the 5th edition of the WHO classification [13]. Patients with HLH caused by other triggers (e.g., hematological malignancy other than aggressive LBCL, infection, rheumatism immunity diseases), with HLH onset after the treatment of hematological malignancy, or with significant missing clinical and follow-up data were excluded. The flowchart for screening enrolled patients was detailed in Fig. 1. This study was approved by the Ethics Committee of the first affiliated hospital of Zhejiang University and conducted in accordance with the Declaration of Helsinki. Informed consent was waived due to the retrospective study design and the absence of actual intervention for the enrolled patients.

### Date collection

Baseline data at HLH diagnosis were collected. Clinical characteristics included age and sex; medical history; clinical manifestations such as fever, hepatosplenomegaly and lymphadenopathy; laboratory parameters including complete blood count, fibrinogen, ferritin, serum triglyceride (TG), lactate dehydrogenase(LDH), albumin, globulin, alanine aminotransferase (ALT), aspartate aminotransferase (AST), total bilirubin (TBIL), soluble interleukin-2 receptor (sIL-2R or sCD25), natural killer (NK) cell activity, cytokines, presence of haemophagocytosis in bone marrow or spleen; Epstein-Barr virus DNA (EBV-DNA) copy number in peripheral whole-blood; histopathology and lymphoma staging.

### Treatment and response assessment

All patients received the first-line anti-HLH treatment according to the international guidelines for the treatment of HLH-2004 [12] and guidelines for the diagnosis and treatment of hemophagocytic syndrome in China (2022 edition). The first-line anti-HLH treatments included the following three strategies: ruxolitinib combined with corticosteroid (Ru-D), etoposide and corticosteroid (E-D) and corticosteroid only (D). Ruxolitinib 15 mg orally administered twice daily until the start of anti-lymphoma treatment or death. Most patients immediately received rituximab-based chemotherapy once diagnosed with lymphoma. The first-line anti-lymphoma regimens included RCHOP (Rituximab, Cyclophosphamide, Doxorubicin/pegylated liposomal doxorubicin, Vincristine and Prednisone), ECHOP (Etoposide combined with CHOP), RCOP (RCHOP without doxorubicin/pegylated liposomal doxorubicin), RECOP (RCOP combined with Etoposide), ECOP (Etoposide, Cyclophosphamide, Vincristine and Prednisone), and RD (Rituximab and Dexamethasone).

Treatment responses of HLH, included complete response (CR) and partial response (PR), were assessed based on criteria from previous studies [14]. Overall response rate (ORR) was calculated as the proportion of patients achieving CR and PR. Failure to achieve PR was classified as no response (NR). OS was defined as the interval between HLH diagnosis and death from any causes or the last follow-up for living patients.

### Statistical analysis

All statistical analyses were performed using SPSS version 26.0 for Windows and GraphPad Prism version 9.5. Baseline characteristics were shown as percentage for categorical variables and medians with range (quartiles) for continuous variables. Categorical variables and continuous variables were compared by the Fisher’s exact test and the *t*-test, respectively. The survival curves were performed using Kaplan-Meier method, with differences between groups analyzed using log-rank test. Cox’s proportional hazard model with forward selection was used to find out independent risk factors of mortality. All variables with *P* values of less than 0.05 in univariate analyses were enrolled in further multivariate analysis. The optimal cutoff values of continuous variables except age were determined by receiver operating characteristic (ROC) curve analysis. We categorized continuous variables according to the cutoff values. Differences with the two-sided *P* values < 0.05 were considered to be statistically significant.

## Results

### Patient characteristics

Clinical characteristics of the patients at diagnosis are detailed in Table 1. Median age of all patients was 60 years (range, 24 to 85 years), with 53.3% being ≥ 60 years (16/30), and the sex ratio was 1:1. NK-cell activity and sIL-2R were evaluated in 13 patients. All patients met at least 5 of 8 HLH-2004 diagnostic criteria, including fever (27/30, 90.0%), splenomegaly (30/30, 100%), cytopenias affecting at least 2 of 3 lineages (29/30, 96.7%), hypertriglyceridemia (7/30, 23.3%), hypofibrinogenemia (4/30, 13.3%), hemophagocytosis in bone marrow (26/30, 86.7%), ferritin more than 500ng/ml (29/30, 96.7%), low NK-cell activity (8/13, 61.5%), and elevated sIL-2R (13/13, 100%). Among 10 patients who underwent whole exome sequencing, 3 were found to harbor rare heterozygous mutations in primary HLH genes, i.e., *UNC13D*, *LYST* and *STXBP2*, respectively. In the cytokine profile of this cohort at HLH diagnosis, serum IL-6 and IL-10 levels were significantly elevated, with median levels of 100.37pg/ml and 991.05pg/ml, respectively.

**Table 1:** Baseline characteristics of the study population

Characteristics, *n*(%) or median (IQR)	All (*n* = 30)	Controlled HLH group (*n* = 17)	Uncontrolled HLH group (*n* = 13)	*P*-value
Gender				0.713
Male	15(50)	8(47.1)	7(53.8)	
Female	15(50)	9(52.9)	6(46.2)	
Age, years	60(53–69)	59(49–68)	67(56–74)	0.143
Fever	27(90)	16(94.1)	11(84.6)	0.565
Splenomegaly	30(100)	17(100)	13(100)	–
Hepatomegaly	5(16.7)	2(11.8)	3(23.1)	0.628
Neutrophils < 1,000/uL	4(13.3)	2(11.8)	2(15.4)	1
Hgb < 90 g/L	29(96.7)	17(100)	12(92.3)	0.433
PLT < 30,000/uL	8(26.7)	2(11.8)	6(46.2)	**0.049**
TG > 3 mmol/L	7(23.3)	3(17.6)	4(13.3)	0.666
Fibrinogen < 1.5 g/L	4(13.3)	2(11.8)	2(15.4)	1
Ferritin > 500 ng/mL	29(96.7)	17(100)	12(92.3)	0.433
Haemophagocytosis	26(86.7)	15(88.2)	11(84.6)	1
sIL-2R > 6400pg/ml^*^	13(100)	10(100)	3(100)	–
Low NK cell activity^*^	8(61.5)	6(60)	2(66.7)	1
LDH > 250 U/L	28(93.3)	16(94.1)	12(92.3)	1
ALT > 40 U/L	10(33.3)	5(29.4)	5(38.5)	0.705
AST > 40 U/L	14(46.7)	6(35.3)	8(61.5)	0.153
Albumin < 30 g/L	22(73.3)	14(82.4)	8(61.5)	0.242
HScore ≥ 169	27(90.0)	15(88.2)	12(92.3)	1
TBiL > 26umol/L	6(20.0)	1(5.9)	5(16.7)	0.061
EBV-DNA positive	8(26.7)	2(11.8)	6(46.2)	**0.049**
Cytokines, pg/mL				
IL-2	0.47(0.1–1.35)	0.12(0.1–1.3)	0.81(0.1–1.63)	0.375
IL-4	0.84(0.1–1.98)	0.98(0.1–1.57)	0.69(0.5–1.98)	0.460
IL-6	100.37(52.06-171.21)	115.6(63.28-187.55)	61.54(43.32-107.68)	0.286
IL-10	991.05(530.67-2349.3)	919.29(475.85-4457.26)	1042.74(704.31-1342.08)	0.884
IL-17 A	0.1(0.1-19.94)	0.1(0.1-15.43)	0.1(0.1–24.6)	0.440
TNF-α	2.64(1.29–4.1)	2.68(1.28–4.49)	2.59(1.74–3.82)	0.950
IFN-γ	8.21(3.66–59.21)	16.36(4.26-134.18)	6.44(2.84–20.18)	0.174

^*^ NK-cell activity and sIL-2R were evaluated in 13 patients

Abbreviations: *Hgb* hemoglobin, *PLT* platelet, *TG* triglyceride, *sIL-2R* soluble interleukin-2 receptor, *LDH* lactic dehydrogenase, *ALT* alanine aminotransferase, *AST* aspartate aminotransferase, *TBiL* total bilirubin. Normal reference range by the clinical laboratory: NK cell activity ≥ 15.11%, IL-2 ≤ 4.1 pg/mL, IL-4 ≤ 3.2 pg/mL, IL-6 ≤ 2.9 pg/mL, IL-10 ≤ 5.0 pg/mL, IL-17 A ≤ 2.9 pg/mL, TNF-α ≤ 23 pg/mL, IFN-γ ≤ 18 pg/mL

All patients were diagnosed with LBCL shortly after their HLH diagnosis. Among these patients, 23 were confirmed by bone marrow biopsy, 7 cases by lymph node biopsy, 4 cases by spleen needle biopsy or splenectomy, 1 case by liver puncture biopsy, and 1 case by adrenal mass puncture biopsy. There were 6 patients who underwent biopsies at two sites, with 5 diagnosed through bone marrow biopsy and lymph node biopsy, and 1 diagnosed through bone marrow biopsy and spleen biopsy. The median time interval from HLH diagnosis to lymphoma diagnosis was 8.5 days (range, 1 to 42 days). Seventeen patients were diagnosed with diffuse large B-Cell lymphoma (DLBCL), including 15 non-germinal center B-cell (non-GCB) and 2 GCB subtypes by the Hans classification using CD10, Bcl-6 and MUM1 immunohistochemical stains. One patient was diagnosed with EBV-positive diffuse large B-cell lymphoma with CD20 negativity and another with intravascular large B-cell lymphoma. Additionally, among 17 patients ultimately diagnosed only by bone marrow examination, 11 patients could not be classified into the subtypes of aggressive LBCL due to the suboptimal quality of the biopsy sample with only a few tumor cells to be found, which made it impossible to obtain enough information of immunohistochemical staining. All patients underwent a fluorodeoxyglucose (FDG) positron emission tomography (PET) -CT scan for staging. Except for 2 cases involving only the spleen, defined as stage IS, all other cases were classified as stage IV according to the Ann Arbor staging system. Eleven cases initially presented with lymphoma involvement only in bone marrow, liver and/or spleen (BLS-type), without lymphadenopathy or involvement of other extranodal organs. Eight patients were tested positive for whole-blood EBV-DNA, with a median of 1800 (range 516–865,000) IU/mL. Only 1 of 26 patients tested for EBER in pathological examinations was positive.

### Anti-HLH treatment and response

**Fig. 2:**
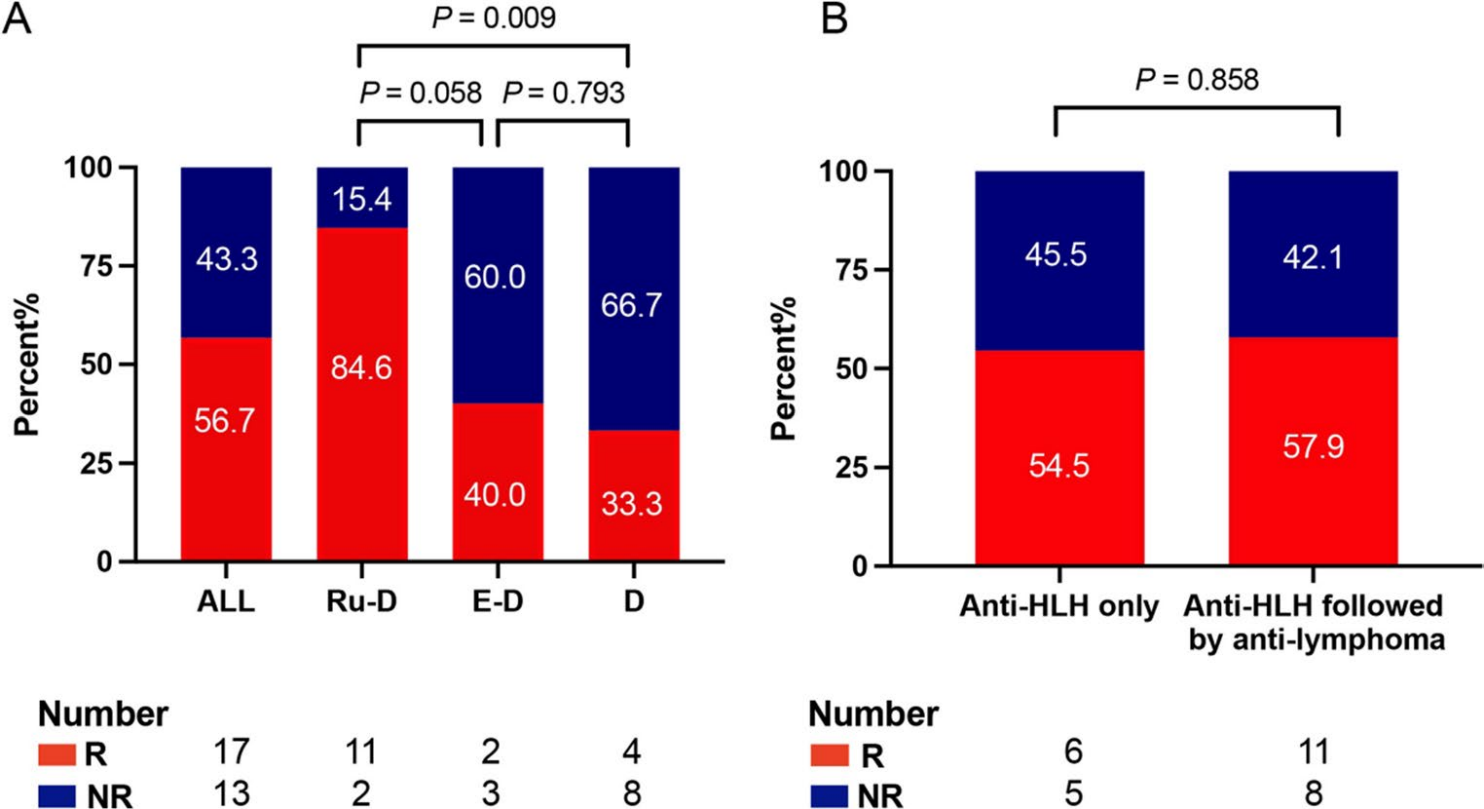
Response outcomes of anti-HLH frontline therapy. **A** Overall response rate (ORR) within 2 weeks in the all patients, ruxolitinib combined with corticosteroid (Ru-D) group, etoposide and corticosteroid (E-D) group and corticosteroid only (D) group. **B** ORR in patients received only anti-HLH therapy and anti-HLH therapy followed by anti-lymphoma therapy group within 2 weeks. R, response (patients who achieved PR or better); NR, no response (patients who failed to achieve PR at least)

Twenty-one (70%) patients initiated anti-HLH treatment within 24 h after diagnosis of HLH (range, 0 to 5 days). In the total cohort, 13 (43.3%) patients received ruxolitinib combined with corticosteroid (Ru-D), 12 (40.0%) received corticosteroid only (D), and 5 (16.7%) received etoposide and corticosteroid (E-D) as first-line treatment for HLH. The median duration of ruxolitinib treatment was 13 days (range: 3–35 days). The 2-week ORR of HLH was 56.7%, with all responding patients achieving only PR. Patients who received Ru-D regimen as first-line treatment for HLH achieved the highest ORR of 84.6%, which was significantly higher than that of 33.3% in the D group (*P* = 0.009) and 40.0% in the E-D group (*P* = 0.058), as shown in Fig. 2A. Eleven patients received only anti-HLH therapy within 2 weeks, achieving an ORR of 54.5%. Similarly, 19 patients who switched from anti-HLH therapy to anti-lymphoma chemotherapy after being diagnosed with lymphoma achieved a comparable ORR of 57.9% (*P* = 0.858, Fig. 2B).

We divided all patients into controlled HLH group and uncontrolled HLH group based on the treatment efficacy of HLH within 2 weeks, and then compared their clinical characteristics. We defined controlled HLH group as patients achieved PR or better within 2 weeks. In the uncontrolled HLH group, the proportions of severe thrombocytopenia (< 30,000/uL) and whole-blood EBV-DNA positivity were significantly higher compared with the controlled HLH group (*P* = 0.049 and *P* = 0.049, respectively). There were no significant differences for all other factors between two groups, as detailed in Table 1. Only 25% of patients with severe thrombocytopenia received the Ru-D regimen as first-line anti-HLH treatment, which was lower than the 50% of patients with platelets ≥ 30,000/uL (*P* = 0.019). When comparing patients with positive and negative EBV-DNA in whole blood, no significant differences were found in the selection of first-line anti-HLH treatment (*P* = 0.076).

### Anti-lymphoma treatment and response

Twenty-seven patients were treated with the first-line anti-lymphoma chemotherapy immediately upon diagnosis. Most patients received a regimen combining rituximab with chemotherapy. Due to the complexity and severity of the condition in these patients, chemotherapy dosages were customized based on each patient’s conditions. To be specific, 8 patients were initially treated with low-intensity chemotherapy regimens without anthracyclines, including 1 case of RCOP, 5 cases of RECOP, 1 case of ECOP and 1 case of RD. Among them, 5 patients subsequently received at least 4 cycles of RCHOP regimen, while the remaining 3 patients underwent lymphoma progression and uncontrolled HLH rapidly, with short survival times of 9 days, 44 days, and 82 days, respectively. In addition, 18 patients received RCHOP regimen, with 2 cases receiving a combination therapy with etoposide. One patient was administered with ECHOP regimen without rituximab due to CD20 negativity. Fifteen patients completed at least 4 cycles of chemotherapy. In contrast, the remaining 3 patients did not receive chemotherapy; one patient passed away on the day of lymphoma diagnosis, and the other two patients refused chemotherapy due to the severity of their illness.

### Outcomes

**Fig. 3:**
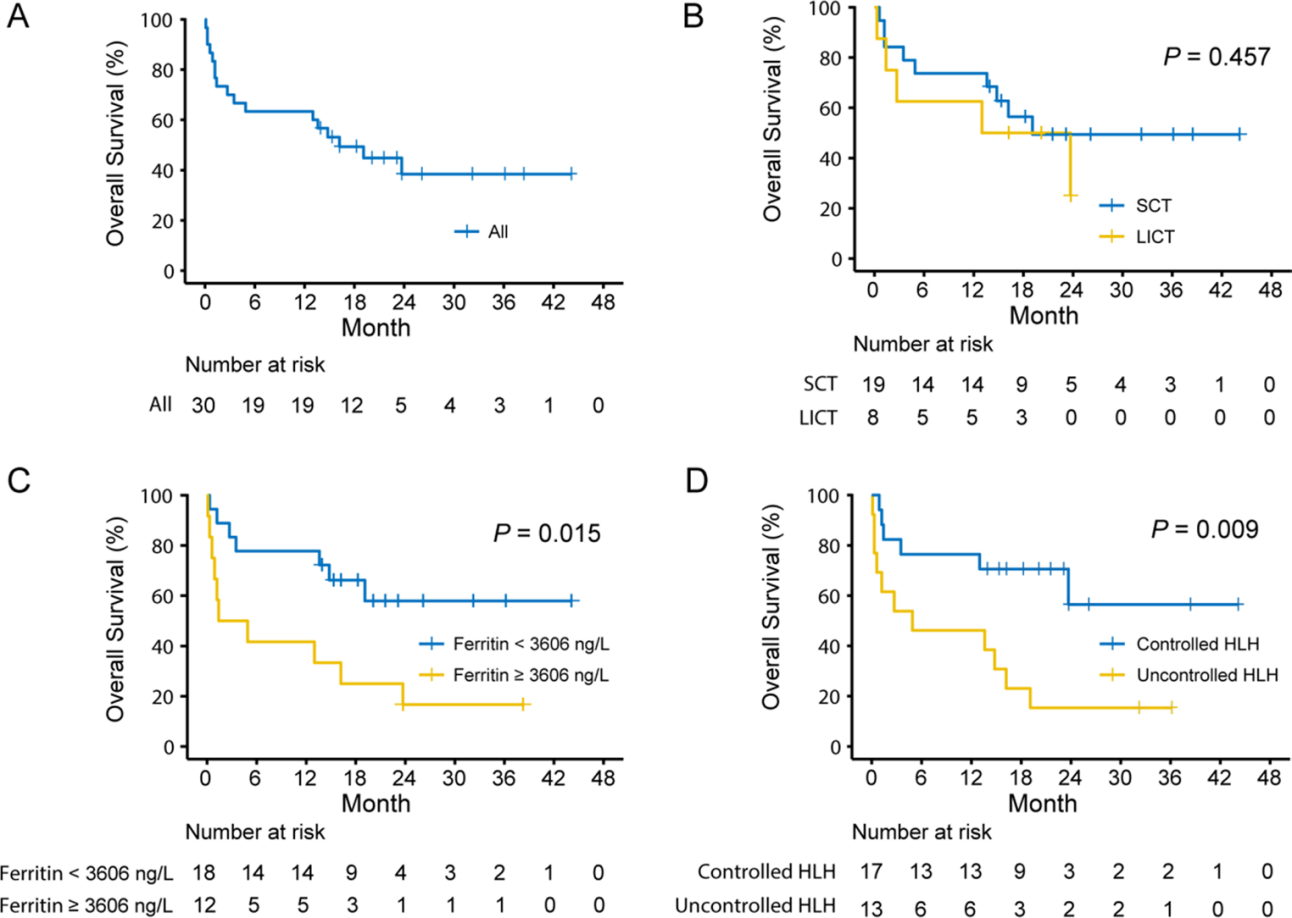
Kaplan-Meier analysis of overall survival (OS). **A** The OS of the whole cohort. Log-rank test was used to compare the differences between groups according to **B** First-line anti-lymphoma chemotherapy (SCT, standard chemotherapy; LICT, low-intensity chemotherapy); **C** Ferritin level; **D** Treatment response of HLH within 2 weeks (Controlled HLH, achieved PR or better; Uncontrolled HLH, failed to achieve PR at least)

The median follow-up time for all patients was 23.7 months (range, 0.1 to 44.1 months). Seventeen patients (17/30, 56.7%) died within the study period, and the early mortality rate within 8 weeks was 26.7% (8/30). The median OS was 16.2 months (95% CI: 7.766–24.634), with a 1-year OS rate of 63.3% and a 2-year OS rate of 38.4% (Fig. 3A). For 11 patients with BLS-type LBCL, the median OS was 19.1 months (95% CI: 11.598–26.602), with a 2-year OS rate of 35.4%. Eight patients did not receive standard-dose chemotherapy after lymphoma diagnosis but were given low-dose chemotherapy without anthracycline, as previously described. The median OS of these patients was 13 months, shorter than the 19.1 months for patients who received standard chemotherapy directly, but the difference between the two groups was not statistically significant (*P* = 0.457, Fig. 3B). Compared with the standard chemotherapy group, the patients in the low-intensity chemotherapy group tended to be older (median age: 69 years vs. 57 years, *P* = 0.079) and have a higher proportion of liver dysfunction (50% vs. 26.3%, *P* = 0.375), but there were no statistical differences. For 20 patients who received at least 4 cycles of RCHOP chemotherapy, the median OS was not reached, with the 1-year OS rate of 95% and the 2-year OS rate of 57.5%. Among them, three patients underwent autologous hematopoietic stem cell transplantation (auto-HSCT). One patient experienced disease relapse at 6 months post-transplantation and died within 3 months after the relapse. The other two patients were still alive at the last follow-up.

### Risk factors associated with mortality

The results of Cox’s proportional hazard analysis of risk factors associated with mortality in patients diagnosed with LBCL accompanied by HLH were shown in Table 2. Univariate analysis showed that age ≥ 60 years, severe thrombocytopenia (< 30,000/uL), ferritin levels ≥ 3606 ng/mL, EBV-DNA positive and uncontrolled HLH within 2-week were associated with poorer OS. Further multivariate analysis showed that ferritin levels ≥ 3606 ng/mL (adjusted HR 3.968, 95% CI 1.468–10.73; *P* = 0.007) and uncontrolled HLH within 2 weeks (adjusted HR 4.415, 95% CI 1.573–12.391; *P* = 0.005) were the independent risk factors associated with inferior OS. Kaplan-Meier survival curves stratified by the independent risk factors were shown in Fig. 3C and D. Patients with ferritin levels ≥ 3606 ng/mL exhibited a very poor survival, with a median OS of 1.4 months (95% CI: 0.0-7.68) and a 1-year OS rate of 16.7%. For the patients with uncontrolled HLH within 2 weeks, the median OS was 4.9 months (95% CI: 0.0-19.462), with a 1-year OS rate of 46.2% and a 2-year OS rate of 15.4%.

**Table 2:** Univariate and multivariate analysis of risk factors associated with mortality in patients diagnosed with LBCL accompanied by HLH

Characteristics	Univariate analysis		Multivariate analysis	
	Hazard ratio (95% CI)	*P*-value	Hazard ratio (95% CI)	*P*-value
Age ≥ 60 years	3.028(1.062–8.63)	0.038	–	0.257
Male	1.126(0.431–2.94)	0.808		
Hemoglobin < 66 g/L	1.767(0.62–5.036)	0.287		
Neutrophils < 1500/uL	3.552(0.809–15.6)	0.093		
Platelet < 30,000/uL	3.111(1.15–8.42)	0.025	–	0.438
Ferritin ≥ 3606 ng/mL	3.125(1.18–8.273)	0.022	3.968(1.468–10.73)	0.007
EBV-DNA positive	3.914(1.455–10.525)	0.007	–	0.144
IL-10 ≥ 888 pg/mL	2.879(0.926–8.949)	0.068		
IL-6 ≥ 100 pg/mL	0.616(0.234–1.626)	0.328		
Uncontrolled HLH within 2 weeks^*^	3.491(1.285–9.479)	0.014	4.415(1.573–12.391)	0.005
Used etoposide within 4 weeks	0.595(0.225–1.572)	0.295		
Low-intensity chemotherapy^#^	1.509(0.505–4.51)	0.461		

Abbreviations: ^*^Patients failed to achieve PR at least within 2 weeks; ^#^First-line anti-lymphoma chemotherapy regimens without anthracyclines

## Discussion

In the present study, we described a series of aggressive LBCL with concomitant HLH at initial diagnosis which are extremely rare and have fulminant clinical course. The majority of patients in this study presented with an advanced-stage disease (IV) and had bone marrow involvement. Our results indicate that prompt and effective control of HLH could significantly improve the overall survival of patients. Among various regimens used in this study, the Ru-D regimen demonstrated the highest efficacy and is thus recommended as the initial anti-inflammatory therapy of HLH. Ferritin levels ≥ 3606 ng/mL and uncontrolled HLH within 2 weeks were the independent risk factors associated with poorer OS.

Patients with HLH always present with fever, cytopenia, and splenomegaly, which may be caused by HLH itself, or secondary to underlying diseases or a combination of both. Due to the lack of specific clinical manifestations and rapid progression, the diagnosis of HLH and its underlying causes can be easily obscured. Hematological malignancies (HM), especially non-Hodgkin lymphomas (NHL), are the most common causes of secondary HLH in adults, accounting for 56% of cases [15]. Zoref-Lorenz et al. developed an improved index comprising ferritin > 1000 µg/L and sCD25 > 3900 U/mL, termed the optimized HLH inflammatory (OHI) index, which accurately identifies HM-associated HLH with best sensitivity and specificity [16]. In our previous retrospective study [17], we identified an effective diagnostic index for lymphoma-associated HLH, with weighted risk scores of 1 assigned to splenomegaly and IL-6 level (≥ 55.1pg/mL) respectively, and a score of 3 assigned to IL-10 level (≥ 425.9pg/mL). When HLH patients have a diagnostic index ≥ 2 points, LAHS should be considered. Based on these diagnostic index, we could preliminarily assess the probability of lymphoma in adult patients with HLH and guide further interventions for early diagnosis of lymphoma.

Aggressive LBCL with concomitant HLH at diagnosis always presents with symptoms such as fever, hepatosplenomegaly, vomiting, nausea, diarrhea, and thrombocytopenia [6, 8, 18–22]. The diagnosis of LBCL in these cases is often challenging, due to the poor detection of lymphadenopathy using physical examination or imaging, and thus the diagnosis is primarily made through bone marrow biopsy. In this study, 17 patients were ultimately diagnosed only by bone marrow examination, and 11 patients initially presented with lymphoma involvement solely in bone marrow, liver and/or spleen (BLS-type), without lymphadenopathy or other mass lesions. Although BLS-type DLBCL is not currently recognized in the World Health Organization (WHO) classification, a few studies have highlighted its unique clinical characteristics [6, 23]. Compared with DLBCL-NOS, BLS-type DLBCL more frequently exhibits activated B-cell (ABC) subtype, a high Ki-67 proliferative index, MYC overexpression, presence of HLH, expression of programmed death ligand 1 (PD-L1) and poor OS [6, 23]. RCHOP regimen, as the current first-line standard therapy for most patients of DLBCL, only yields an 18% of 2-year OS rate for BLS-type DLBCL cases [6]. The survival of BLS-type patients in our study was still poor, with 2-year OS rate of 35.4%. It is necessary to explore more effective treatment strategies for BLS-type patients, especially in those with concomitant HLH.

Regardless of the diverse causes of HLH, the common terminal pathway of its pathogenesis is the activation of T cells and macrophages, leading to uncontrolled secretion of proinflammatory cytokines. However, the mechanisms of pathogenesis in aggressive LBCL-associated HLH remain unclear. It may be related to continuous antigen stimulation and the release of pro-inflammatory cytokines by the lymphoma cells, EBV infection, unbalanced gene rearrangements or inherited immune defects [1, 7, 24]. Repeated stimulation of Toll-like receptor 9 (TLR9) or myeloid differentiation factor 8 (MYD88) also promotes HLH progression, as evidenced in mouse models [25, 26]. Silencing MYD88 inhibited antigen presenting cell (APC) maturation, hyperactivation of CD8^+^ T cells, and cytokine production [26]. In DLBCL, the ABC subtype is more likely to accompany HLH than the GCB subtype. MYD88 L265P mutation presents in 29% of patients with DLBCL ABC subtype. This mutation leads to activation of the NF-κB pathway and the subsequent activation of CD8^+^ T cells to produce proinflammatory cytokines, which may contribute to the development of HLH [27]. In a genetic analysis study, DLBCL patients with HLH showed more frequent rearrangements and additions in the immune-related gene loci (such as 19q13 encoding IFN-γ or 9p24 encoding PD-L1) compared with those without HLH [7].

Treatment for LBCL-associated HLH varies depending on patients’ clinical conditions and physician’s opinion. Currently, there is still no standard recommended treatment strategy. For patients with HLH as the initial presentation of an undiagnosed lymphoma, the primary treatment goal is to treat HLH with an appropriate anti-inflammatory therapy based on the severity and urgency of the disease, while creating favorable conditions for the subsequent diagnosis and treatment of lymphoma. Our data also confirmed that patients who responded to anti-HLH therapy within 2 weeks had significantly better OS than non-responsive group. The Ru-D regimen was the most effective treatment with an ORR of 84.6%, superior to the etoposide-based protocol. However, all patients who responded to the Ru-D regimen only achieved PR. Therefore, timely diagnosis of lymphoma is crucial, as early intervention of anti-lymphoma chemotherapy is key to prevent HLH from progressing rapidly. However, intensive chemotherapy can be fatal, due to severe cytopenia or organ dysfunction secondary to HLH and lymphoma. Thus, upon lymphoma diagnosis, the selection of first-line chemotherapy should balance efficacy with adverse effects. Our result indicates that low-intensity chemotherapy without anthracycline as the first-line of anti-lymphoma therapy followed by RCHOP or ECHOP did not compromise survival. Patients treated with low-intensity chemotherapy were older and had a higher proportion of liver dysfunction. Therefore, for older patients or those with compromised conditions or multi-organ dysfunction, a low-intensity chemotherapy regimen can be adopted as a transitional strategy during initial anti-lymphoma treatment to balance efficacy with treatment side effects, with the potential to switch to standard chemotherapy once HLH is controlled and organ functions improve.

Recently, some studies have explored the diagnostic and prognostic value of sCD25 and ferritin level in HM-HLH, but the cutoff levels are still unclear. In patients with HM-HLH, the OHI index included sCD25 > 3900 U/mL and ferritin > 1000 µg/L is associated with a high mortality risk [16]. In a recent systematic scoping review, sCD25 levels > 10,000 U/mL and ferritin levels > 15,000 µg/L predicted poor prognosis for OS in lymphoma-associated HLH [28]. Ferritin elevation in B-NHL associated HLH was less prominent compared with T-NHL, with a median value of 2785 µg/L, and ferritin > 15,000 µg/L was only seen in 18% (16/89) of B-NHL cases [28]. In our cohort, the median ferritin was 3168 µg/L, with only 2 patients exceeding 15,000 µg/L, both of whom died within two months. Ferritin ≥ 3606 µg/L was an independent risk factor associated with poorer OS. sCD25 was significantly elevated in all 13 patients tested in the present study, with a median value of 37,562 (range 14,712 − 204,465) pg/ml. Their cutoff values to predict prognosis vary greatly, which may be related to the different types of HM and various treatment strategies.

The main limitation of our study is the small number of cases. However, as LBCL-HLH is a rare disease, it is difficult to collect large sample cases in a single center. Also, its retrospective nature may introduce information and recall bias. Finally, certain data were incomplete, such as the absence of c-Myc and Bcl-2 rearrangement information, making further confirmation of high-grade B-cell lymphoma impossible.

In conclusion, our study demonstrated that patients with aggressive LBCL accompanied by HLH at diagnosis have a high early mortality and short survival period. The primary treatment goal for these patients is to promptly and effectively control HLH activity, followed by a rapid identification of lymphoma. Ru-D regimen may be recommended as the preferred treatment strategy. In some critically ill patients, low-intensity chemotherapy without anthracycline may be considered as the first-line chemotherapy to balance the disease severity with treatment side effects. In addition, our data reinforce the necessity of exploring the underlying molecular mechanisms of LBCL with concomitant HLH at diagnosis and developing prospective clinical trials to find more effective treatment strategies.

## Data Availability

No datasets were generated or analysed during the current study.
